# Supplementary material for: Predictive Value of Clinical and Dual-Energy Computed Tomography Parameters for Hemorrhagic Transformation and Long-Term Outcomes Following Endovascular Thrombectomy
Source: Diagnostics (Basel). 2024 Nov 19;14(22):2598. doi: 10.3390/diagnostics14222598 (PMC11592779; doi:10.3390/diagnostics14222598)
Supplement: Supplementary file 1 [file diagnostics-14-02598-s001.zip › diagnostics-3299164-supplementary.pdf]

Supplementary Table S1. Logistic Regression Analysis of Comorbidities for Predicting Hemorrhagic Transformation

	Simple model		
	OR	(95% CI)	p value
Hypertension	1.04	(0.52- 2.09)	0.914
Diabetes Mellitus	0.51	(0.26- 1.03)	0.060
Dyslipidemia	1.06	(0.52- 2.16)	0.871
Atrial fibrillation	1.25	(0.65- 2.43)	0.507
Coronary artery disease	1.14	(0.48- 2.67)	0.768
Peripheral artery occlusion disease	0.85	(0.26- 2.82)	0.794
Previous stroke history	1.20	(0.50- 2.91)	0.683
Previous usage of antiplatelet	0.61	(0.26- 1.40)	0.241
Previous usage of anticoagulant	1.73	(0.67- 4.43)	0.257
Previous tPA	0.65	(0.33- 1.27)	0.206

Logistic regression. *$p<0.05$, **$p<0.01$.

Supplementary Table S2. Non-Parametric Analysis of Additional Variables for In-Hospital Mortality

	No (n=139)		Yes (n=13)		p value
	Median	IQR	Median	IQR	
post-EVT 24hr NIHSS	12.00	(7-18)	19.00	(13-33)	0.003**
Gender, n (%)					0.134
Female	62	(44.60%)	3	(23.08%)	
Male	77	(55.40%)	10	(76.92%)	
Hypertension, n (%)	93	(66.91%)	10	(76.92%)	0.550
Diabetes Mellitus, n (%)	44	(31.65%)	5	(38.46%)	0.757
Dyslipidemia, n (%)	99	(71.22%)	7	(53.85%)	0.214
Atrial fibrillation, n (%)	84	(60.43%)	5	(38.46%)	0.124
Coronary artery disease, n (%)	23	(16.55%)	5	(38.46%)	0.065
Peripheral artery occlusion disease, n (%)	11	(7.91%)	1	(7.69%)	1.000
Previous stroke history, n (%)	26	(18.71%)	0	(0%)	0.126
Previous usage of antiplatelet, n (%)	24	(17.27%)	3	(23.08%)	0.703
Previous usage of anticoagulant, n (%)	25	(17.99%)	0	(0%)	0.128
Previous tPA, n (%)	54	(38.85%)	7	(53.85%)	0.291
TOASTT, n (%)					0.012*
CE	94	(67.63%)	5	(38.46%)	
LAA	21	(15.11%)	1	(7.69%)	
Unknown or Other	24	(17.27%)	7	(53.85%)	
Age	71.00	(63-79)	67.00	(59.5-77.5)	0.640
BMI (kg/m2)	24.09	(21.7-26.3)	22.95	(22-26.2)	0.635
WBC	8790.00	(7070-11170)	9400.00	(6045-11885)	0.836
Hb	13.70	(12-15)	13.70	(11.7-15.1)	0.833
Platelet	196.00	(161-237)	209.00	(153-248.5)	0.937
HbA1c	5.90	(5.5-6.4)	6.10	(5.6-7.2)	0.296
Cholesterol	155.50	(130.8-181.5)	146.00	(114-180)	0.271
Triglyceride	72.00	(55-103.8)	103.00	(82-193.3)	0.004**
HDL	47.00	(41-58)	37.00	(29-44)	0.002**
LDL	90.50	(73-113.3)	79.50	(64.3-112.3)	0.360
PT	10.85	(10.5-11.4)	11.30	(10.5-12.3)	0.306
APTT	26.25	(24.6-28.3)	25.30	(23.7-29.6)	0.701
ER NIHSS	17.00	(13-21)	21.00	(16-27.5)	0.091
DECT condition, n (%)					0.246
DECT-NCCT(-)	75	(53.96%)	5	(38.46%)	
IOM(+)VNC(-)	11	(7.91%)	3	(23.08%)	
IOM(-)VNC(+)	24	(17.27%)	3	(23.08%)	
IOM(+)VNC(+)	29	(20.86%)	2	(15.38%)	
Hyperdense area on sNCCT(+) volume (mm3)	2895.00	(1063.8-7953.8)	2847.50	(1232.5-19597.5)	0.734
Hyperdense area on sNCCT(+) density (HU)	45.50	(38.3-53.8)	46.50	(39.5-51.8)	0.809
IOM(+) area iodine concentration (mg/ml)	0.83	(0.6-1.3)	1.20	(0.8-1.5)	0.178

IOM(+) area density (HU)	48.20	(41.5-59.5)	47.90	(45.4-62)	0.598
Caudate nucleus iodine concentration (mg/ml)	0.26	(0.2-0.3)	0.30	(0.2-0.3)	0.231
Caudate nucleus density (HU)	35.30	(33.9-37.1)	36.20	(32-37.4)	0.854
Superior saggital sinus iodine concentration (mg/ml)	0.31	(0.2-0.4)	0.33	(0.2-0.7)	0.534
Superior saggital sinus density (HU)	43.10	(39.3-46.6)	43.90	(41.2-54.4)	0.267
MRI, n (%)	122	(87.77%)	11	(84.62%)	0.667
Ischemic volume(mm³) by MRI	16590.00	(4681.5-47220)	15006.00	(5406-156198)	0.756
Ischemic Area(mm²) by MRI	27.65	(7.8-78.7)	25.01	(9-260.3)	0.756
VNC ASPECT score	7.00	(6-8)	7.00	(5.8-9)	0.687

Mann-Whitney U test. Chi-Square test. Fisher exact test.
